# Quantified, Interactive Simulation of AMCW ToF Camera Including Multipath Effects

**DOI:** 10.3390/s18010013

**Published:** 2017-12-22

**Authors:** David Bulczak, Martin Lambers, Andreas Kolb

**Affiliations:** Computer Graphics Group, Institute for Vision and Graphics, University of Siegen, 57076 Siegen, Germany; martin.lambers@uni-siegen.de (M.L.); andreas.kolb@uni-siegen.de (A.K.)

**Keywords:** time-of-flight, sensor simulation, BRDF

## Abstract

In the last decade, Time-of-Flight (ToF) range cameras have gained increasing popularity in robotics, automotive industry, and home entertainment. Despite technological developments, ToF cameras still suffer from error sources such as multipath interference or motion artifacts. Thus, simulation of ToF cameras, including these artifacts, is important to improve camera and algorithm development. This paper presents a physically-based, interactive simulation technique for amplitude modulated continuous wave (AMCW) ToF cameras, which, among other error sources, includes single bounce indirect multipath interference based on an enhanced image-space approach. The simulation accounts for physical units down to the charge level accumulated in sensor pixels. Furthermore, we present the first quantified comparison for ToF camera simulators. We present bidirectional reference distribution function (BRDF) measurements for selected, purchasable materials in the near-infrared (NIR) range, craft real and synthetic scenes out of these materials and quantitatively compare the range sensor data.

## 1. Introduction

Amplitude modulated continuous wave Time-of-Flight (AMCW-ToF) depth sensors provide per-pixel distance information by estimating the phase shift of a received amplitude modulated light signal that has been emitted by an active light source using a reference signal [1]. This phase shift is proportional to the time light traveled from the light source to the sensor. Despite recent rapid development progress, AMCW ToF cameras still suffer from several error sources. Some of the major effects that severely influence AMCW ToF range measurements are motion artifacts, flying pixels and multipath interference (MPI).

The simulation of AMCW ToF cameras including reproduction of the major sensor effects benefits the development of new sensors by allowing tests of variations to the sensor design [2], as well as the development of down-stream data processing algorithms by providing ground truth and test data [3,4]. AMCW ToF simulation requires modeling the illumination, the light propagation in the scene, and the individual sensor pixel behavior. Furthermore, computationally efficient approaches are of great importance for simulating dynamic scenes and/or for parameter studies in hardware layout and algorithm design [2,5].

Up to now, there has only been very little research in multipath interference (MPI) simulation for AMCW ToF cameras. Meister et al. simulate MPI using *non-interactive* global illumination schemes, which implicate very high computational costs of approximately 2 h per depth image, including simple analytic bidirectional reflectance distribution functions (BRDFs) [4]. Furthermore, there is very little research in providing *quantitative* comparison to *real-world* measurements, which is indispensable to reliably predict the behavior of prospective AMCW ToF cameras and their application. Such a comparison needs to take real scene material properties into account, typically provided by BRDF measurements. We are not aware of any work that uses such measurements at the relevant near-infrared wavelength for AMCW ToF simulation.

In this paper, we present a physically based simulation method for AMCW ToF cameras that runs on interactive frame rates. Our approach is based on Lambers et al. [2], which already accounts for physical units. Our simulation approach is fully GPU-based and comprises the following contributions:

- Enhancement of the *Reflective Shadow Map (RSM)* algorithm [6] for GPU-based, *interactive, single-bounce, image-space, multipath interference* simulation.
- *BRDF-based reflection simulation* for measured real-world materials.
- Extension of the simulation model to include realistic electronic and optical shot noise.

Furthermore, this paper presents evaluation approaches for ToF simulations based on data captured by real cameras with the following contributions:

- Measurement of isotropic BRDF at 850 nm wavelength for several specified materials that can be purchased worldwide and thus can be used to reproduce scenes reliably.
- Quantitative evaluations of the proposed simulator based on AMCW ToF camera acquisition of real-world reference scenes. We clearly see the improved ToF simulation results of our single-bounce approach over direct simulation in terms of quality, and over higher-order global illumination simulation [4] in terms of computational performance.
- Publicly available simulator, BRDF data including references to material vendors, geometry of the reference scenes, and real AMCW ToF camera measurements, in order to promote further activities in quantitative evaluation of AMCW ToF simulation.

## 2. Related Work

Keller and Kolb [5] present a GPU-based AMCW ToF simulation approach that computes light propagation in real-time by using basic rasterization techniques, including local illumination with a Lambertian reflection model. Their simulation approach generates so-called *phase images*, i.e., the raw images acquired by a AMCW-ToF camera, and can reproduce spatio-temporal artifacts such as flying pixels and motion artifacts.

Meister et al. [4] propose an AMCW ToF simulation method that adopts a global illumination technique, i.e., bidirectional path tracing, to simulate multipath interference. This approach is computationally very expensive and only suitable to simulate static scenes. They provide a visual comparison with real data on range image basis for two scenes (“corner” and “box”) as well as limited quantitative comparison of simulated data with real data captured with a PMDTec CamCube 3 (pmd technologies ag, Siegen, Germany). Neither real material properties are acquired nor used within their simulator.

Alternative AMCW ToF sensor simulation approaches have a stronger focus on the sensor hardware. Schmidt and Jähne [7] model optical excitation and target response to simulate the conversion of photons to electrical charges. Their approach does not simulate light propagation and illumination.

Lambers et al. [2] introduce a realistic sensor model to simulate both the photometric relations in the scene including light propagation and illumination, and physically correct charges at a sensor pixel’s readout circuit level, that result from incoming photons. Their simulation is limited to scene materials that are Lambertian reflectors. They provide quantitative comparison of AMCW ToF camera simulation data with real captured data, but their evaluation is limited to sensor pixel based plausibility checks of their simulation model and ignores characteristic error sources.

So far, none of the existing AMCW ToF simulation approaches is capable of simulating MPI effects at interactive rates, none take realistic scene material properties into account in the light propagation simulation, and no quantitative evaluation is available at range image level.

Ritschel et al. [8] give an overview of interactive global illumination methods. These approximations of global illumination are more suitable to simulate MPI effects at interactive rates than the more general, but computationally much more expensive methods, such as the bidirectional path tracing used by Meister. Image-space approximations of global illumination such as Reflective Shadow Maps [6] are especially efficient and sufficient for our use case despite their limitations.

In addition to global illumination approximation, realistic scene materials need to be taken into account to allow comparisons of simulated results and measurements. Most existing BRDF databases for material properties, such as the widely known MERL [9] and CUReT [10] databases, focus on visible wavelengths and are typically limited to RGB channels.

Lacking near-infrared BRDF measurements, Mutny et al. [11] use an Oren–Nayar BRDF model with parameters fitted from the CUReT database for AMCW ToF simulation using Meister’s simulator to create a database of scenes for correcting multipath artifacts based on a machine learning approach.

Choe et al. [10] recently published the first BRDF database for near-infrared wavelengths, demonstrating that material properties at these wavelengths may differ significantly from those at visible wavelengths. They focus on finely-structured materials such as fabrics. In contrast, in this work, we focus on standardized materials that are available for purchase worldwide, in order to allow reliable reproduction of scenes with defined material properties.

## 3. Time-of-Flight Simulation

Our simulation model is based on Lambers et al. [2] that consists of two parts, the direct light propagation (Section 3.1) and sensor pixel behaviour (Section 3.2). We describe our extension of this model with respect to BRDF-based materials (Section 3.1), multipath effect simulation via global illumination approximation (Section 3.3), and a realistic noise model (Section 3.4). For further information on the AMCW ToF principle, we refer the reader to, e.g., [1,2,12].

### 3.1. Direct Light Propagation

**Figure 1:**
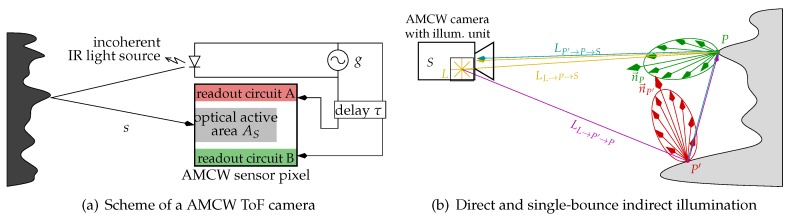
Scheme of a AMCW ToF camera including the pixel layout and the optically active pixel area $A_{S}$ (gray) with the two readout circuits *A* (red) and *B* (green) (**a**); direct illumination and one path of single-bounce indirect illumination in the AMCW ToF simulation (**b**).

The model described by Lambers et al. [2] assumes Lambertian reflectors only. We directly extend this model to use BRDFs. Starting with the power $P_{L}\left[W\right]$ of the camera light source *L*, we can deduce the radiant intensity $I(\theta_{L\to P})$ from *L* to a surface point *P*. This radiant intensity can be assumed constant for an isotropic light source model, or taken from a vendor-provided intensity table depending on the angle $\theta_{l\to P}$ between the main light direction ${\vec{n}}_{L}$ and P−L; see Figure 1b.

The irradiance $E_{l\to P}$ [W/m${}^{2}$] of *P* resulting from direct illumination is:

(1) $$E_{L\to P}=I_{L}\left(\theta_{L\to P}\right)\frac{\cos\theta_{P\to L}}{{∥P-L∥}^{2}},$$

where $\theta_{P\to L}$ is the angle between L−P and the surface normal ${\vec{n}}_{P}$ at *P*. This notation will be used throughout the following derivations.

The resulting direct-illumination radiance $L_{l\to P\to S}$ from surface point *P* to sensor *S* depends on the material of the surface, described by its BRDF $f_{l\to P\to S}$ at *P* for incoming light direction l→P and outgoing light direction P→S:

(2) $$L_{L\to P\to S}=E_{L\to P}\cdot f_{L\to P\to S}.$$

### 3.2. Sensor Pixel Model

The AMCW ToF sensor consists of an array of w×h sensor pixels. Each sensor pixel accumulates charges in the readout circuits, *A* and *B*, depending on its irradiation; see Figure 1a. The irradiance $E_{S}$ of the pixel’s photosensitive area $A_{S}$ resulting from direct illumination is given as

(3) $$E_{L\to P\to S}=L_{L\to P\to S}\cdot \cos\theta_{S\to P}.$$

$E_{l\to P\to S}$ determines the optical power $P_{S}\left[W\right]$ and from that the energy $W_{S}\left[J\right]$ that is accumulated in one pixel over the integration time *T* for a common AMCW duty cycle of 50%:

(4) $$P_{S}=E_{S}\cdot A_{S},\;W_{S}=P_{S}\cdot T\cdot 0.5.$$

The conversion into electron–hole pairs in the pixel depends on the quantum efficiency $\nu_{q}$, which describes how many electrons are generated per incoming photon, and the wavelength λ:

(5) $$N_{\mathrm{tot}}=\frac{W_{S}}{\nu_{q}\cdot \frac{q\cdot \lambda }{h\cdot c}},$$

where *h* is the Planck-constant, *c* is the speed of light, and *q* is the value of elementary charge.

This total charge $N_{\mathrm{tot}}=N_{A}+N_{B}$ is accumulated in the two circuits *A* and *B* depending on the phase shift ϕ, the internal phase delay τ, and the achievable demodulation contrast D∈[0,1]:

(6) $$N_{A}=\frac{N_{tot}}{2}\left(1+D\cdot f\left(\tau ,\phi \right)\right),\;N_{B}=\frac{N_{tot}}{2}\left(1-D\cdot f\left(\tau ,\phi \right)\right).$$

Here, *f* is the *correlation function* resulting from the mixing of the optical signal *s* with the delayed reference signal *g*; see Figure 1a. Commonly, it is assumed that *g* and *s* are ideal cosine shaped functions, which results in a cosine shaped correlation function f(τ,ϕ)=cos(τ+ϕ).

### 3.3. Multipath Simulation

The simulation model of Lambers et al. [2] is restricted to direct illumination, i.e., light paths l→P→S. Here, we describe an approximation of the total radiance $L_{P\to S}$ reaching the sensor *S* from point *P* based on the rendering equation (see Figure 1b), which in our notation is

(7) $$L_{P\to S}=\int_{P^{\prime }\in \mathrm{scene}}f_{P^{\prime }\to P\to S}\cdot L_{P^{\prime }\to P}\cdot \cos\theta_{P\to P^{\prime }}\cdot V_{P,P^{\prime }}\;dP^{\prime },$$

where $f_{P^{\prime }\to P\to S}$ is the BRDF at *P* for incoming light direction $P^{\prime }\to P$ and outgoing light direction P→S, and $V_{P,P^{\prime }}=1$ if *P* and $P^{\prime }$ are mutually visible, otherwise 0.

We adapt the ideas of instant radiosity [13] and its implementation via Reflective Shadow Maps (RSMs) [6] for our purposes. First, we separate the direct illumination path $L_{l\to P\to S}$ from the single-bounce indirect illumination paths $L_{P^{\prime }\to P\to S}$. Second, we consider a discrete set of points $P^{\prime }$ in the scene that are directly illuminated by *L*. This set of virtual point lights (VPLs) is generated by rendering the scene from the point of view of the light source into a two-dimensional map (called reflective shadow map, RSM). Each pixel in this RSM describes one VPL. Note that the RSM approach ignores multi-bounce indirect illumination paths and realizes single-bounce multipath reflections only.

Considering the separated direct and single-bounce reflections using RSM, the rendering equation then simplifies to

(8) $$L_{P\to S}=L_{L\to P\to S}+\sum_{P^{\prime }\in \mathrm{RSM}}f_{P^{\prime }\to P\to S}\cdot L_{L\to P^{\prime }\to P}\cdot \cos\theta_{P\to P^{\prime }}\cdot V_{P^{\prime },P}\cdot W_{P^{\prime },P}$$

with (analogous to the direct illumination path)

(9) $$L_{L\to P^{\prime }\to P}=E_{L\to P^{\prime }}\cdot f_{L\to P^{\prime }\to P},\;E_{L\to P^{\prime }}=I_{L}\left(\theta_{L\to P^{\prime }}\right)\frac{\cos\theta_{P^{\prime }\to L}}{{∥P^{\prime }-L∥}^{2}}$$

and the VPL weight factor

(10) $$W_{P^{\prime },P}=A_{P^{\prime }}\frac{\cos\theta_{P\to P^{\prime }}}{{∥P^{\prime }-P∥}^{2}}.$$

$W_{P^{\prime },P}$ describes the steradiant of the VLP $P^{\prime }$ (considered as area light source) at surface point *P*. In order to provide all necessary information, each VPL in the RSM stores the irradiance $E_{l\to P^{\prime }}$ and the VPL’s area $A_{P^{\prime }}$.

Furthermore, for the image-space RSM approach, visibility tests between *P* and $P^{\prime }$ are impractical, thus we simplify this term by suppressing light directions below the horizon, i.e.,

(11) $$V_{P,P^{\prime }}=\left\{\begin{array}{cc} 1, & \mathrm{if}\;\theta_{P^{\prime }\to P}<\frac{\pi }{2}\wedge \theta_{P\to P^{\prime }}<\frac{\pi }{2},\\ 0, & \mathrm{otherwise}. \end{array}\right..$$

As the results will show, the limitations of the RSM approach are acceptable in our use case. Its benefits as a purely image-space approach are its simplicity and efficiency, especially for GPU-based implementations.

In contrast to the original RSM approach that samples VLPs only in the vicinity of *P*, we sample the entire RSM when computing the incident radiance at *P*.

In the sensor pixel, the charges $N_{A}$ and $N_{B}$ now result from a superimposed signal from the direct and multiple single-bounce indirect light paths. We convert radiance incident to the sensor pixel from indirect illumination paths and $L_{l\to P^{\prime }\to P\to S}$ into irradiance

(12) $$E_{L\to P^{\prime }\to P\to S}=\cos\theta_{S\to P}\cdot L_{P\to S}$$

and can then deduce electron pair counts $N_{\mathrm{tot},l\to P^{\prime }\to P\to S}$ for each indirect path using Equations (4) and (5).

Denoting the phase shift along the direct and a single-bounce indirect light path as $\phi_{l\to P\to S}$ and $\phi_{l\to P^{\prime }\to P\to S}$, respectively, we can compute the total charges $N_{A}$ and $N_{B}$ by combining Equations (6) and (8), yielding

(13) $$\begin{array}{cc} N_{A}= & \frac{N_{\mathrm{tot},L\to P\to S}}{2}\left(1+D\cos\left(\tau +\phi_{L\to P\to S}\right)\right)+\sum_{P^{\prime }\in \mathrm{RSM}}\frac{N_{\mathrm{tot},L\to P^{\prime }\to P\to S}}{2}\left(1+D\cos\left(\tau +\phi_{L\to P^{\prime }\to P\to S}\right)\right), \end{array}$$

(14) $$\begin{array}{cc} N_{B}= & \frac{N_{\mathrm{tot},L\to P\to S}}{2}\left(1-D\cos\left(\tau +\phi_{L\to P\to S}\right)\right)+\sum_{P^{\prime }\in \mathrm{RSM}}\frac{N_{\mathrm{tot},L\to P^{\prime }\to P\to S}}{2}\left(1-D\cos\left(\tau +\phi_{L\to P^{\prime }\to P\to S}\right)\right). \end{array}$$

### 3.4. Noise Model

Electronic and optical shot noise plays the dominant role for ToF cameras [12], which is especially the case for low light situations [14]. In contrast to other noise sources, shot noise cannot be reduced by signal processing methods but has an impact on range resolution $\Delta L=\frac{L}{360^{\circ }}\cdot \Delta \phi$ with non-ambiguity range *L* and phase error Δϕ [12]. Shot noise is Poisson distributed, but fitting an explicit Poisson model to is known to be numerically unstable. A common approach to handle this instability is to use variance stabilization transformations [15]. We apply a Freeman–Tukey (FT) transform [16], which transforms the Poisson distributed charge values into an approximate standard normal distribution. Applying a Gaussian fitting to the FT-transformed mean and variance values yields the distribution model in FT space.

**Figure 2:**
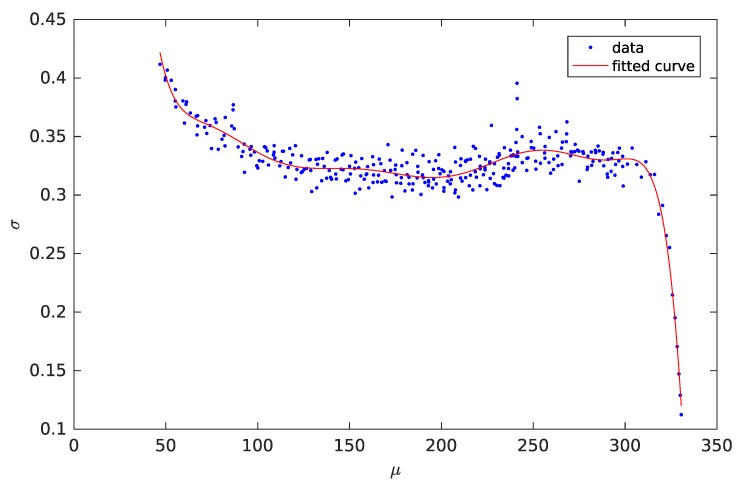
Plot of noise model that describes the Gaussian variance as function of the mean value (transformed intensity) in Freeman–Tukey space with **SSE**=0.0391 and **RMSE** =0.0112.

More precisely, we acquire 1000 images of the ToF camera, a PMD CamCube 3.0 in our case, for 80 different integration times. Correcting each raw image by subtracting the dark image (fix pattern noise) yields the raw data that is transformed into FT space. Selecting random pixels in each batch of 1000 × 4 phase images, mean and variance values are computed (blue dots in Figure 2). In order to deduce the final noise model, we apply a polynomial fitting of degree 9 over the the mean-variance measurements (see Figure 2). The resulting fit error is (sums of squares error) **SSE** =0.0391 and (root mean square error) **RMSE**=0.0112. Applying the model is done by transforming the charge values from Equations (13) and (14) into the FT domain, computing the Gaussian parameters by evaluating the variance curve for the given intensity, generating the noise value using a random number and the variance, and, finally, back-transforming this value in the original domain of the charge values. This noise value is then added to the noise-free charge value in order to get the final charge value.

## 4. NIR BRDF Measurements

In addition to multipath and noise effects in the simulator (Section 3), a quantified comparison between simulated and real AMCW ToF data requires a realistic model of materials in the scene in the form of BRDFs. Analytic BRDF models such as Cook-Torrance [17] offer only limited capabilities to represent real world material. Databases of measured BRDFs, such as MERL [9] and CUReT [10], do not provide data for the operating wavelength of AMCW ToF camera, i.e., around 870 nm, and/or do not contain standardized, purchasable materials that can be used to craft real-world reference scenes.

In this section, we briefly describe the measurement and data processing procedures to acquire isotropic BRDF at 850 nm. We apply this procedure to standard material that can be purchased worldwide (see Section 5).

### 4.1. Measurement Setup

**Figure 3:**
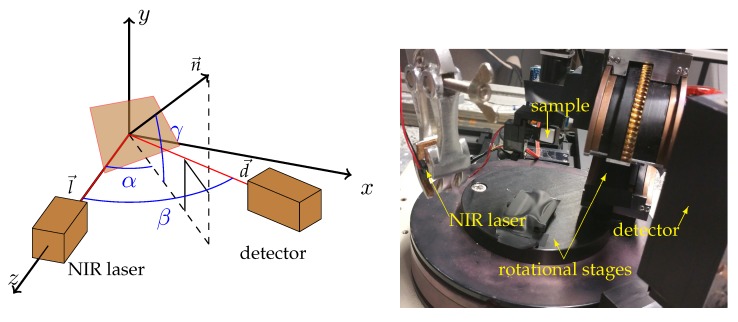
The schematic gonioreflectometer measurement setup (**left**) and a photo of the real setup (**right**).

Even though there are various potential approaches to acquire BRDFs [18], similar to Li et al. [19], we opt for a rather simple approach using a three-axis gonioreflectometer. The gonioreflectometer controls the elevation angles of the incoming and outgoing light and their relative azimuth; see Figure 3 left.

Our measurement setup is parameterized using the angles $\alpha =∠(\vec{n^{\prime }},\vec{l})$, $\beta =∠(\vec{d},\vec{l})$ and the elevation angle γ of the sample’s normal $\vec{n}$. Here, $\vec{n^{\prime }}$ is the projection of $\vec{n}$ onto the $\vec{l}$-$\vec{d}$-plane, $\vec{l}={\left(0,0,1\right)}^{T}$ the light direction towards the NIR laser, and $\vec{d}$ the direction towards the detector; see Figure 3, right. Using a simple vector calculus, we get $\vec{n}={\left(\sin\alpha \cdot \cos\gamma ,-\sin\gamma ,\cos\alpha \cdot \cos\gamma \right)}^{T}$ and $\vec{d}={\left(\sin\beta ,0,\cos\beta \right)}^{T}$. This simply transfers to the angles $\theta_{i},\theta_{o}$ between the surface normal and the light and detector direction, respectively, and the azimuthal difference $\phi_{d}$ with respect to the sample’s coordinates:

(15) $${\left(\theta_{i},\theta_{o},\phi_{d}\right)}^{T}={\left(\arccos\left(\vec{n}\cdot \vec{z}\right),\arccos\left(\vec{n}\cdot \vec{d}\right),\arccos\left(\vec{z^{\prime }}\cdot \vec{d^{\prime }}\right)\right)}^{T},$$

where $\vec{z^{\prime }}$ and $\vec{d^{\prime }}$ are the projections of $\vec{z}$ and $\vec{d}$ onto the plane perpendicular to $\vec{n}$.

### 4.2. Extrapolating BRDF Measurements

Due to physical limitations, the angular regions $\beta \in [-10^{\circ },10^{\circ }]$ and $\theta_{i},\theta_{o}>80^{\circ }$ can not be acquired. Inspired by the work of Panzer and Ponteggia [20], who have a similar problem in sampling directional acoustic reflection values, we tested two approaches: inverse distance weighting (IDW), which is basically a Shepard’s method, and spherical harmonic interpolation. In our experiments, we found that a extended version of the first method delivers the most reliable results.

Given the BRDF acquisition values $f(\vec{\rho^{\prime }})$ for sampling angular parameters $\vec{\rho^{\prime }}=\left(\theta_{i}^{\prime },\theta_{o}^{\prime },\phi_{d}^{\prime }\right)$, the IDW approach computes the BRDF $f(\vec{\rho })$ for an unmeasured parameter vector $\vec{\rho }$ by taking the measurements in its neighborhood N(ρ) into account:

(16) $$f\left(\vec{\rho }\right)=\left\{\begin{array}{cc} f(\rho^{\prime }), & \mathrm{if}\;\rho \to \rho^{\prime }\\ \frac{1}{W}\sum_{\vec{\rho^{\prime }}\in N\left(\vec{\rho }\right)}w\left(\vec{\rho },\vec{\rho^{\prime }}\right)\cdot f\left(\vec{\rho^{\prime }}\right), & \mathrm{else} \end{array}\right.,\;\mathrm{with}\;W=\sum_{\rho^{\prime }\in N\left(\rho \right)}w\left(\rho ,\rho^{\prime }\right).$$

Here, $w(\vec{\rho },\vec{\rho^{\prime }})$ is a distance measure, or weight, between the two parameter vectors. In our case, we define *w* as

(17) $$w\left({\vec{\rho }}_{1},{\vec{\rho }}_{2}\right)={\left(\arccos\left({\vec{l}}_{1}\cdot {\vec{l}}_{2}\right)+\arccos\left({\vec{d}}_{1}\cdot {\vec{d}}_{2}\right)\right)}^{-u}$$

with incoming ${\vec{l}}_{1},{\vec{l}}_{2}$ and outgoing directions ${\vec{d}}_{1},{\vec{d}}_{2}$ corresponding to ${\vec{\rho }}_{1}$ and ${\vec{\rho }}_{2}$, respectively. This weight definition accounts for angular differences between both the incident and the outgoing angles. Thus, measured BRDF values have a large weight in the BRDF estimation of a set of unobservable angular parameters, if their angular parameters are similar. We use u=5, which has been determined experimentally.

## 5. Results

In this section, we present the results related to the BRDF measurement for standard materials (Section 5.1) and the evaluation of the multipath interference using the proposed simulation technique (Section 5.2).

### 5.1. BRDF Measurement

The measurement setup has been equipped with a 850 nm NIR-laser as a light source (LDM850/5LT, Roithner Lasertechnik GmbH, Vienna, Austria) and a photo diode as detector. The chosen sampling stepsizes are $\Delta \alpha =\Delta \beta =\Delta \gamma =2^{\circ }$ for specular material and $\Delta \alpha =\Delta \beta =\Delta \gamma =5^{\circ }$ for diffuse materials. To reduce noise, we averaged 1000 measurements for each parameter set $\vec{\rho }=\left(\alpha ,\beta ,\gamma \right)$. Finally, we normalized the measured reflection values by the maximum energy arriving directly from laser to detector and the cosine of the incident angle $\theta_{i}$ in order to get the final BRDF values.

We have selected materials with rather diffuse and rather glossy reflection properties. Since we consider reflections but not scattering, transmission, and other properties, we have chosen opaque materials so that almost no light transmission occurs. As diffuse materials, we have chosen guttagliss PVC rigid foam variants [21], as materials with glossy specular reflection we have chosen PLEXIGLAS${}^{®}$ [22]; see Table 1.

**Figure 4:**
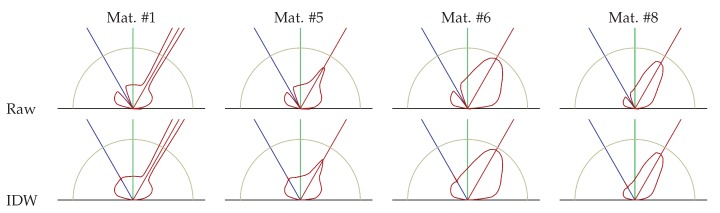
BRDF raw data and IDW interpolation results for materials #1, #5, #6 and #8 acquired for an incident light angle $\theta_{i}=30^{\circ }$. The blue ray indicates the incident light direction, the red ray the ideally reflected incident light direction and the red curve the BRDF value related to the corresponding ray from the center to a point of this curve.

Figure 4 shows the polar plots of the raw measured and the resulting interpolated BRDFs; see Section 4. Plots of the raw measurement visualize the missing measurements around the incoming direction for $\beta \in [-10^{\circ },10^{\circ }]$. The IDW interpolation closes the gap and slightly smooths the measurements.

#### Open Science

Upon acceptance of this paper, we will make the full BRDF data publicly available, thus other researchers can purchase the respective materials in order to setup their own test scenes for quantitative evaluations.

### 5.2. Simulator Evaluation

**Figure 5:**
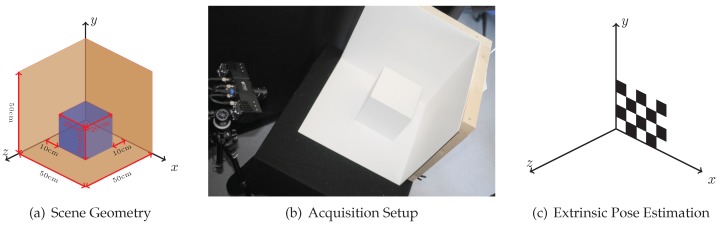
The geometry of our box scene (light brown) with the additional cube (blue) (**a**); a photo of the AMCW ToF measurement setup (**b**); and the positioning of the calibration pattern for ToF camera pose estimation (**c**).

In our evaluation of the simulator, we have used three different scene geometries. All geometries are variants of a corner, for which multipath effects are to be expected (see Figure 5a): **Corner** is a simple corner scene without an additional cube, in the **CornerCube** scene an additional cube is placed directly in the corner, and in the **CornerCubeShift** the cube is shifted by 10 cm from each corner wall. We have setup the three scenes with **Material #1** and **Material #5**.

For real world data acquisition, we have used a PMD CamCube 3.0 ToF camera that captures depth images at 200×200 px resolution. The CamCube’s driver delivers data that is already corrected for the so-called wiggling error, which is a systematic error occurring due to imperfections in the signal shape of the real-world camera with respect to the theoretically assumed cosine function; see Equation (6). Its active light source operates at 870 nm wavelength. The ToF camera is positioned symmetrically towards the corner. Even though there are sophisticated approaches that utilize range and intensity data to determine intrinsic and extrinsic ToF camera parameters [23], we use OpenCV’s implementation Zhang’s simple checkerboard method [24] to estimate these parameters (see Figure 5c). Lindner [25] reports a distance error of less than 0.9–1.9 cm at 1.2–2.2 m using this method (see Table 2.2 in [25]). The range measurements of the PMD CamCube are denoted as **CamCube**, while the simulation results are labeled as **SimDirect** (only direct reflection is simulated) and **SimSingle** (additional single-bounce indirect reflections are simulated). Furthermore, we add the ground truth depth information for comparison (**GroundTruth**). Our evaluation indirectly compares to Lambers et al. [2], as **SimDirect** essentially is the approach in Lambers et al. [2] enhanced with the noise model described in Section 3.4 and BRDF-based reflection (instead of Lambertian).

**Figure 6:**
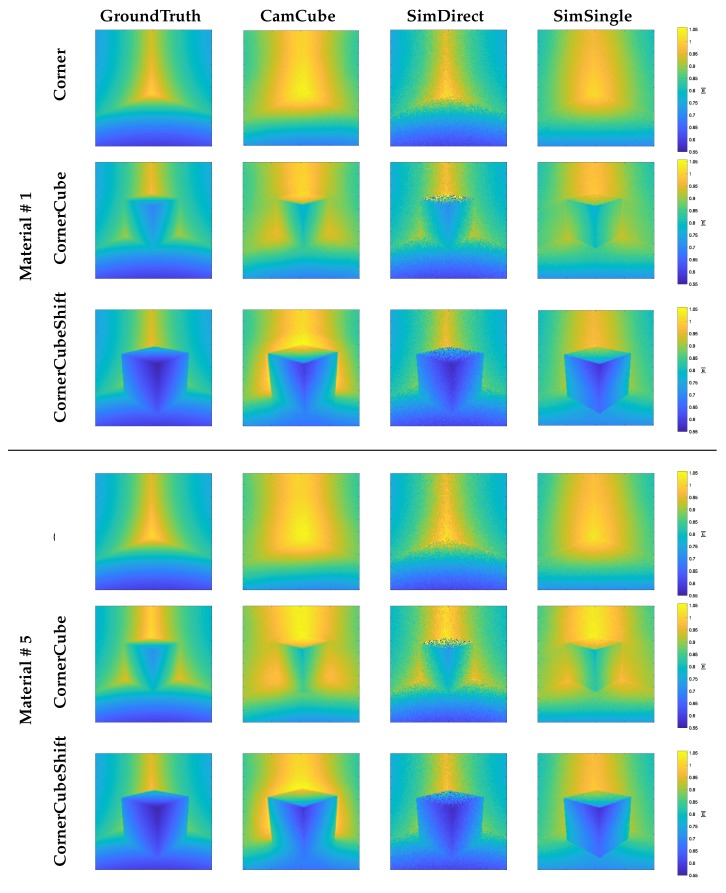
Range image comparison: **GroundTruth** (left) and **CamCube** (mid-left) compared with simulation using direct illumination (**SimDirect**, mid-right) and single bounce reflection (**SimSingle**, right) for the three test scenes **Corner** (rows 1,4), **CornerCube** (rows 2,5) and **CornerCubeShift** (rows 3,6) for **Material # 1** and **Material # 5**.

**Figure 7:**
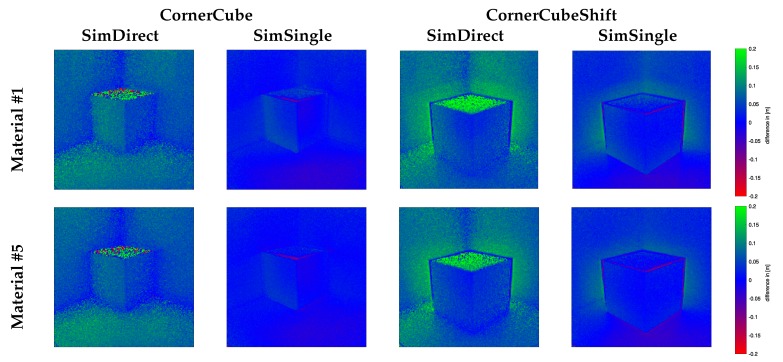
Signed difference images to the **CamCube** measurement for **CornerCube** and **CornerCubeShift**: **GroundTruth** (Left) and the simulation with direct illumination (**SimDirect**, middle) and with single bounce reflection (**SimSingle**, Right) for **Material #1** (top row) and **Material #5** (bottom).

**Figure 8:**
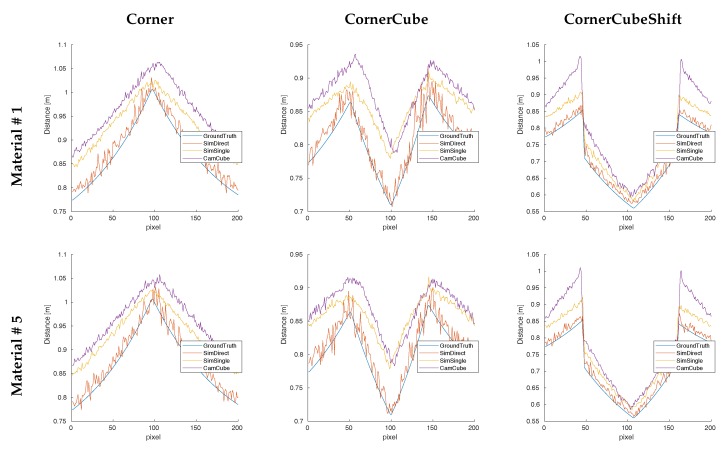
Range comparison for scan lines 100 for the three test scenes **Corner** (**Left**), **CornerCube** (**Middle**) and **CornerCubeShift** (**Right**) for **Material #1 and Material #5**.

Figure 6 shows the depth images for all three test-scenes for **GroundTruth**, the **CamCube** measurements, and the **SimDirect** and **SimSingle** simulations. Figure 7 and Figure 8 give additional insight into range simulation results by showing the signed differences between the simulation and the **CamCube** measurements for the scenes **CornerCube, CornerCubeShift** and the explicit range values along row 100 for all three scenes, respectively. As expected, the real ToF data exhibits significant multipath effects in all three scenes. Considering the simulation without multipath component (**SimDirect**), the resulting range values are close to the ground truth depth. This is consistent with the ToF measurement principle, which explicitly considers direct reflection only. Table 2 states all error values for all scenes and material with respect to the measured **CamCube** data. Especially for scenes **Corner** and **CornerCube**, our approach outperforms **SimDirect** since it captures multipath effects in the corners. In **CornerCubeShifted**, our approach still decreases the errors by more than 50%. In summary, we find that adding single-bounce indirect reflections (**SimSingle**) significantly improves the simulation results with respect to the **CamCube** measurements. This is especially the case for the **Corner** and the **CornerCube** scenes. For the **CornerCubeShift** scene, however, the deviation between the ToF measurement **CamCube** and the simulation including single-bounce reflections **SimSingle** still deviate, mainly in the visual corners between the base corner and the inserted, shifted cube.

**Figure 9:**
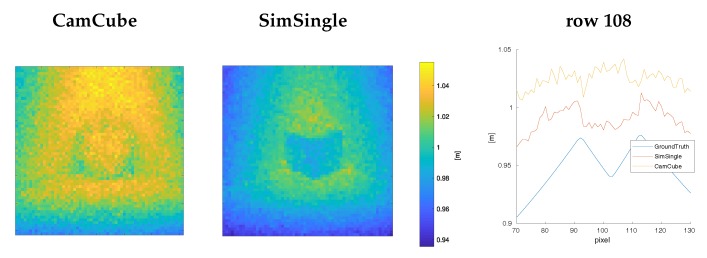
Evaluation of the corner scene with an aluminum cube (extraction of pixel regions [70,130]×[70,130]): this scene comprises a significantly larger amount of multipath effects that cannot be fully covered by our single-bounce simulation method.

Figure 9 shows the limitations of our simulation method. We have used a variant of the **CornerCube** scene, where have placed an aluminum cube with an edge length of 5 cm into the glossy corner (**Material #1**). In our simulation, we have used a Cook-Torrance BRDF to model the reflection behavior of the aluminum cube. In this highly reflective scenario, the multipath effects have a strong influence on the **CamCube** measurements (see range values in row 108, Figure 9 right). Here, the cube nearly vanishes in the distance measurements between pixels 90 and 110. **SimSingle**, in comparison, cannot capture this camera behavior, as no higher order multipath effects are simulated.

Regarding the noise model, we observe that the noise level of the **SimSingle** simulations and the real ToF measurements **CamCube** are comparable, whereas the noise level for the direct simulation **SimDirect** is higher. This is due to the fact that the total amount of charge is lower and that, in this case, the additive Poisson noise is with a fixed amplitude. Thus, the relative impact of the noise is higher in the case of the lower overall charge in the **SimDirect** simulation. This effect gets very apparent for flat incident angle with respect to the direct light–surface impact .

Some final notes on the performance of the simulator: The computation of RSMs takes approx. 1.5 ms. Since we sample the whole RSM, the final accumulation step takes approx. 80 ms. Thus, our simulation operates at interactive frame rates. Meister et al. [4], the only other AMCW simulation method accounting for multipath effects, requires computation times in the order of hours.

## 6. Conclusions

We present an enhancement of a physically-based simulation technique for AMCW ToF cameras with respect to multi-path effects and shot noise, while maintaining interactive frame rates in a GPU-based implementation. We further provide a database of BRDF measurements in the near-infrared range for a selection of purchasable materials. This database enables researchers to build up their own real-world and virtual reference scenes out of materials with known reflection properties. This allows for quantitative comparison between corresponding real-world and virtual scenes and, thus, allows for quantitative evaluation of ToF cameras.

The comparison of simulated and measured depth data in Section 5 demonstrates that the combination of BRDF measurements, multi-path effects, and noise enables realistic simulation results that closely match real measurements of reference scenes, even though our model contains single-bounce indirect reflections only.

In future work, it would be interesting to adapt our simulation technique to other types of sensors, e.g., pulse-based ToF cameras, which are also affected by multi-path effects. These cameras operate at similar wavelengths so that our BRDF database again facilitates quantitative comparisons of simulated and real data.

## Figures and Tables

**Table 1:** Materials used for BRDF measurement. The PLEXIGLAS${}^{®}$ provides more specular reflection, and the PVC rigid foam is rather diffuse.

Mat. No.	PLEXIGLAS${}^{®}$ (Glossy)	Mat. No.	Guttagliss PVC (Diffuse)
1	XT (allround), White WN297 GT	5	Rigid Foam, White
2	XT (allround), Red 3N570 GT	6	Rigid Foam, Red
3	XT (allround), Green 6N570 GT	7	Rigid Foam, Green
4	XT (allround), Blue 5N870 GT	8	Rigid Foam, Blue
		9	Rigid Foam, Yellow
		10	Rigid Foam, Gray

**Table 2:** Evaluation of error for all simulation methods and scenes with respect to to the measured **CamCube** data. For each method you can see the the mean-absolute-error (**MAE**), mean-squared-error, (**MSE**) and the root-mean-squared-error (**RSME**).

		Corner		CornerCube		CornerCubeShifted	
		Material #1	Material #5	Material #1	Material #5	Material #1	Material #5
**GroundTruth**							
	**MAE**	0.1001	0.0998	0.0831	0.0790	0.0969	0.0897
	**MSE**	0.0103	0.0105	0.0071	0.0068	0.0106	0.0091
	**RMSE**	0.1017	0.1025	0.0853	0.0823	0.1029	0.0956
**SimDirect**							
	**MAE**	0.0885	0.0884	0.0728	0.0688	0.0846	0.0777
	**MSE**	0.0084	0.0085	0.0079	0.0069	0.0086	0.0074
	**RMSE**	0.0916	0.0924	0.0888	0.0829	0.0927	0.0859
**SimSingle**							
	**MAE**	0.0238	0.0200	0.0194	0.0138	0.0396	0.0342
	**MSE**	0.0007	0.0005	0.0006	0.0003	0.0023	0.0017
	**RMSE**	0.0270	0.0226	0.0233	0.0171	0.0476	0.0417

## References

[1] Kolb A., Barth E., Koch R., Larsen R. (2010). Time-of-Flight cameras in computer graphics. Comput. Graph. Forum.

[2] Lambers M., Hoberg S., Kolb A. (2015). Simulation of Time-of-Flight sensors for evaluation of chip layout variants. IEEE Sens..

[3] Nair R., Meister S., Lambers M., Balda M., Hofmann H., Kolb A., Kondermann D., Jähne B. (2013). Ground truth for evaluating time of flight imaging. Time-of-Flight and Depth Imaging. Sensors, Algorithms, and Applications.

[4] Meister S., Nair R., Kondermann D. (2013). Simulation of Time-of-Flight sensors using global illumination. Vision, Modeling &amp Visualization.

[5] Keller M., Kolb A. (2009). Real-time simulation of time-of-flight sensors. Simul. Model. Pract. Theory.

[6] Dachsbacher C., Stamminger M. Reflective shadow maps. Proceedings of the Symposium on Interactive 3D Graphics and Games.

[7] Schmidt M., Jähne B. (2009). A physical model of time-of-flight 3D imaging systems, including suppression of ambient light. Dynamic 3D Imaging.

[8] Ritschel T., Dachsbacher C., Grosch T., Kautz J. (2012). The state of the art in interactive global illumination. Comput. Graph. Forum.

[9] Matusik W., Pfister H., Brand M., McMillan L. (2003). A Data-Driven Reflectance Model. ACM Trans. Graph..

[10] Choe G., Narasimhan S.G., So Kweon I. Simultaneous estimation of near IR BRDF and fine-scale surface geometry. Proceedings of the IEEE 2016 IEEE Conference on Vision and Pattern Recognition (CVPR).

[11] Mutny M., Nair R., Gottfried J.M. (2015). Learning the correction for Multi-Path deviations in Time-of-Flight Cameras. arXiv.

[12] Lange R., Seitz P. (2001). Solid-state time-of-flight range camera. IEEE J. Quantum Electron..

[13] Keller A. Instant radiosity. Proceedings of the Conference on Computer Graphics and Interactive Techniques (SIGGRAPH).

[14] Conde M.H., Zhang B., Kagawa K., Loffeld O. (2016). Low-light image enhancement for multiaperture and multitap systems. IEEE Photonics J..

[15] White G.C., Bennetts R.E. (1996). Analysis of frequency count data using the negative binomial distribution. Ecology.

[16] Freeman M.F., Tukey J.W. (1950). Transformations related to the angular and the square root. Ann. Math. Stat..

[17] Schlick C. (1994). An Inexpensive BRDF Model for Physically-based Rendering. Comput. Graph. Forum.

[18] Achutha S. (2006). BRDF Acquisition with Basis Illumination; Chapter 2.2 “BRDF Acquisition”. Ph.D. Thesis.

[19] Li H., Foo S.C., Torrance K.E., Westin S.H. (2006). Automated three-axis gonioreflectometer for computer graphics applications. Opt. Eng..

[20] Panzer J., Ponteggia D. (2011). Inverse Distance Weighting for Extrapolating Balloon-Directivity-Plots.

[21] All-Foam Products Company. http://products.allfoam.com/.

[22] Evonik Cyro LCC. http://www.plexiglas-shop.com/.

[23] Lindner M., Schiller I., Kolb A., Koch R. (2010). Time-of-Flight Sensor Calibration for Accurate Range Sensing. Comput. Vis. Image Underst..

[24] Zhang Z. (2000). A flexible new technique for camera calibration. IEEE Trans. Pattern Anal. Mach. Intell. (PAMI).

[25] Lindner M. (2010). Calibration and Realtime Processing of Time-of-Flight Range Data. Ph.D. Thesis.

